# Fishing of α-Glucosidase’s Ligands from *Aloe vera* by α-Glucosidase Functionalized Magnetic Nanoparticles

**DOI:** 10.3390/molecules26195840

**Published:** 2021-09-26

**Authors:** Hao Yuan, Hong Wan, Yi-Kao Hu, Emmanuel Ayodeji Ayeni, Qiang Chang, Chao Ma, Xun Liao

**Affiliations:** 1 Chengdu Institute of Biology, Chinese Academy of Sciences, Chengdu 610041, China; yuanhao18@mails.ucas.ac.cn (H.Y.); wanhong@medaxis.cn (H.W.); 18788184755@126.com (Y.-k.H.); ayeniemmanuel11@mails.ucas.ac.cn (E.A.A.); changqiang21@mails.ucas.ac.cn (Q.C.); 2 University of Chinese Academy of Sciences, Beijing 100049, China; 3 Phytochemistry Laboratory, Tibet Plateau Institute of Biology, Lhasa 850001, China

**Keywords:** *Aloe vera*, α-glucosidase, ligand fishing, magnetic nanoparticles

## Abstract

α-Glucosidase was immobilized on magnetic nanoparticles (MNPs) for selective solid-phase extraction of the enzyme’s ligands present in *Aloe vera*, which is a medicinal plant used for the treatment of various diseases and possesses anti-diabetic activity. One new compound, aloeacone (**2**), together with two known compounds, aloenin aglycone (**1**) and aloin A (**3**), were fished out as the enzyme’s ligands. The structure of **2** was determined by HR-MS and comprehensive NMR techniques. Compound **3** exhibited a weak inhibitory effect on α-glucosidase, while compounds **1** and **2** were found to possess activation effects on the enzyme for the first time. It is interesting that both an inhibitor and agonists of α-glucosidase were fished out in one experiment.

## 1. Introduction

Diabetes is a metabolic disorder that results in the inability of the body to produce or respond to insulin, leading to increased glucose levels in the blood. It is the third leading disease around the world and causes a major public health threat [1]. It was reported that one out of eleven adults has diabetes, and about 90% of those cases were linked to type 2 diabetes (T2D) [2]. α-glucosidase is the key enzyme for regulating blood glucose, which has been widely recognized as a target of anti-diabetes drugs [3]. Several α-glucosidase inhibitors were developed into drugs such as acarbose, miglitol, and voglibose to treat diabetes [4]. However, these drugs were reported to have some side effects such as bloating, intestinal spasms, and abdominal pains [5]. Therefore, it is necessary to discover new inhibitors of the enzyme for design and development of new anti-diabetes drugs.

*Aloe vera*, belonging to the family of Liliaceae, is a well-known pharmaceutical plant that has long been used worldwide to treat various diseases [6]. Yongchaiyudha et al. investigated the effect of *A. vera* juice on diabetes patient to find that it could decrease fasting blood-glucose (FBG) and triglycerides levels [7]. Huseini et al. studied the effects of *A. vera* leaves gel on T2D patients, showing that it lowered the blood-glucose level without adverse effects [8]. Jong-Anurakkun and co-workers isolated aloeresin A from the methanol extract of *A. vera,* which exhibited inhibitory activity against rat intestinal sucrase and maltase, with IC_50_ values of 11.9 and 2.2 mM, respectively [9]. Aloin A is one of the major components present in *A. vera*, which was reported to be an α-glucosidase inhibitor with IC_50_ value of 0.34 mg/mL [10]. Further, chysalodin was isolated from the plant as α-glucosidase’s inhibitor with IC_50_ value of 13.4 ± 1.5 μM [11]. In our systematic screening of anti-diabetic traditional Chinese medicinal plants, the extract of *A. vera* was found to have relatively weak inhibitory activity. This result was supported by Indrianingsih and co-workers reporting that the α-glucosidase inhibitory activity of *A. vera* extract was 8.2% at 200 μg/mL [12]. It is worth further screening active components responsible for the anti-diabetic activity of *A. vera*. Traditional screening methods for active natural products mainly rely on bioactivity-guided chromatographic separation, which is time-consuming and expensive. In recent years, affinity solid-phase extraction (also called ligand fishing) based on the specific binding between receptor and ligand has been widely used in the field of analytical chemistry [13,14,15], among which magnetic solid-phase extraction (MSPE) is specially fit for screening of natural products. Magnetic materials such as the oxides of iron, cobalt, nickel, and other metals are good adsorbents for MSPE, while Fe_3_O_4_ is the most widely used one due to its good biocompatibility and large specific surface area [16,17].

In this work, we screened the ligands of α-glucosidase present in the extract of *A. vera* by developing a facile ligand fishing method based on α-glucosidase immobilized magnetic nanoparticles (AG–MNPs) combined with HPLC-MS. The ligands of α-glucosidase fished out were identified, and their effects on α-glucosidase were assayed. Moreover, the binding modes between the ligands and α-glucosidase were studied. Especially, the new compound (**2**) was directly enriched by the ligand fishing method, and its structure was determined by HR-MS as well as 1D- and 2D-NMR.

## 2. Results

### 2.1. Characterization of AG-MNPs

**Figure 1:**
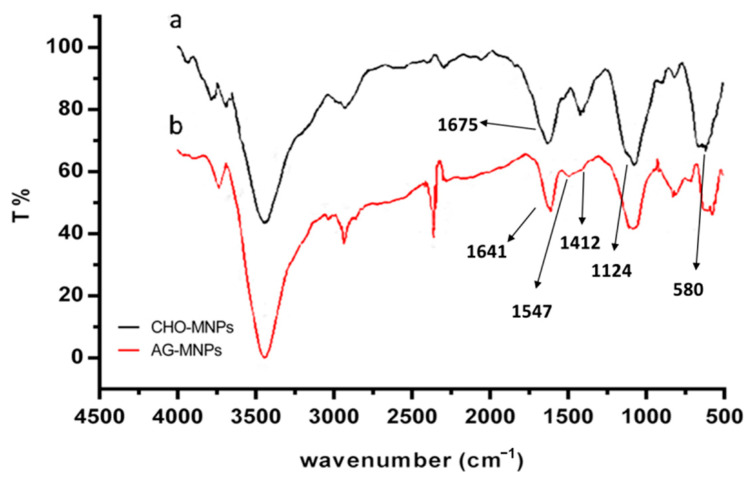
FT-IR spectrum of (**a**) CHO-MNPs, (**b**) AG-MNPs.

AG-MNPs were synthesized by immobilizing α-glucosidase on the aldehyde groups’ functionalized MNPs (CHO–MNPs). The FT-IR spectra of CHO-MNPs and AG-MNPs are shown in Figure 1. The strong IR band at 580 cm^−1^ in Figure 1a was characteristic of the Fe-O vibrations, and the one at 1124 cm^−1^ was ascribable to the asymmetric linear vibration of Si–O–Si bond, indicating the formation of SiO_2_-coated MNPs. The characteristic absorption peak of C=O stretching vibration at 1675 cm^−1^ suggested that the CHO-MNPs were synthesized successfully. In Figure 1b, the peaks at 1412, 1547, and 1641 cm^−1^ were ascribable to peptide bond –NH–CO–, i.e., C-N stretching, N-H deformation, and C=O stretching vibration, respectively, suggesting that α-glucosidase was successfully immobilized on the MNPs.

**Figure 2:**
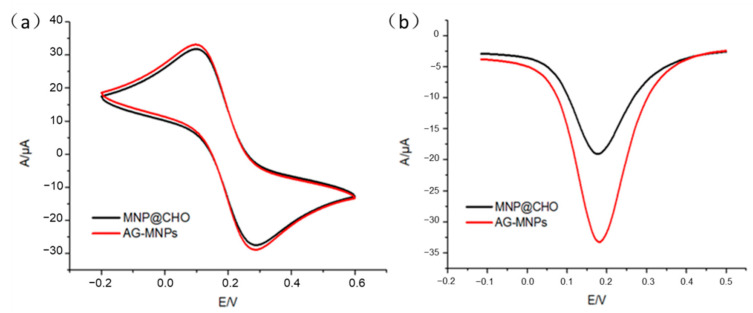
(**a**) Cyclic voltammograms (CV) of CHO-MNPs and AG-MNPs; (**b**) differential pulse voltammograms (DPV) of CHO-MNPs and AG-MNPs.

As shown in Figure 2a, stable and significant REDOX peaks of AG-MNPs and CHO-MNPs, i.e., 103 and 282 mV at the cathode and the anode points, were observed in the cyclic voltammograms (CV) measurement. After introduction of α-glucosidase onto the surface of CHO-MNPs, the peak currents were increased accordingly. The increase of the peak currents for the AG-MNPs was caused by the covalent binding between α-glucosidase and CHO-MNPs, which can weaken the repulsion of aldehyde groups with [Fe(CN)_6_]^3−/4−^, making the conductivity of MNPs dominant. The results of CV reflected that α-glucosidase was immobilized on the surface of CHO-MNPs successfully. In addition, differential pulse voltammograms (DPV) curves also support the above conclusion in that the peak currents (Figure 2b) were increased with the subsequent modifications of α-glucosidase on the surface of CHO-MNPs.

### 2.2. Screening and Structural Classification of α-Glucosidase Ligands from Extract of A. vera

**Figure 3:**
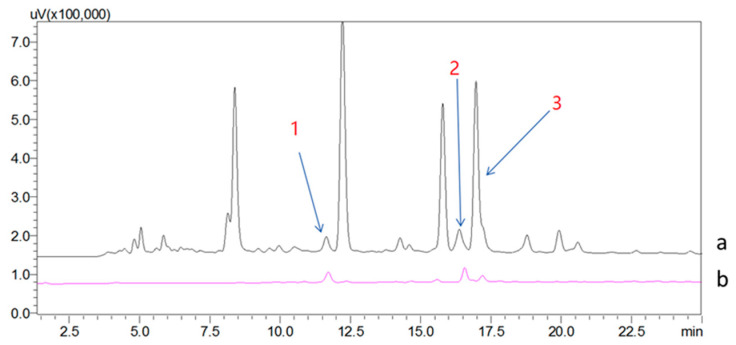
The chromatograms of (**a**) the *A. vera* extract (S_0_) and (**b**) the 50% ACN eluent from AG-MNPs (S_5_).

AG-MNPs were used as the solid phase extraction adsorbent to enrich the ligands of α-glucosidase from the extract of *A. vera*. The HPLC chromatograms of *A. vera* extract (S_0_) and the 50% ACN eluate from AG-MNPs (S_5_) are shown in Figure 3. It is noted that there were more than fifteen compounds detected in S_0_, but only three of them were observed in S_5_, which were denoted as compounds **1**, **2**, and **3**.

**Figure 4:**
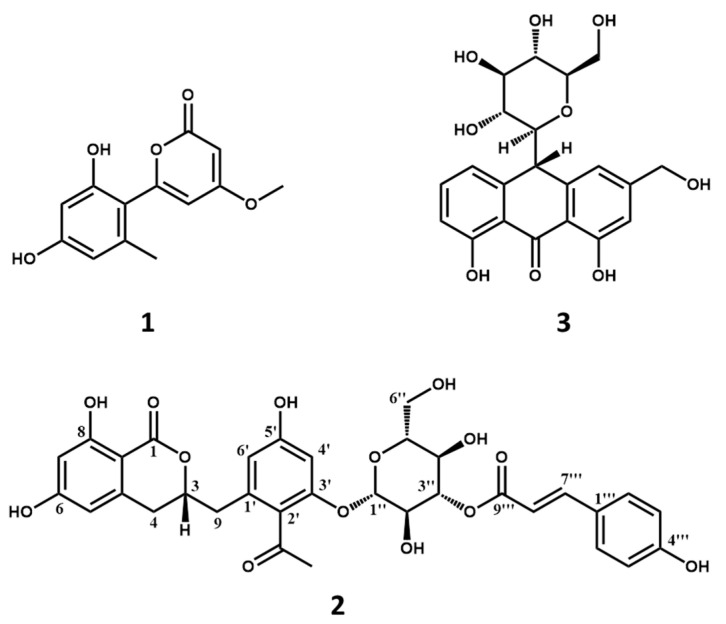
Chemical structures of compounds **1**, **2**, and **3**.

Compounds **1** and **3** possessed molecular weights of 248 (*m*/*z* 247, (M − H)^−^) (Figure S1) and 418 (*m*/*z* 419, (M + H)^+^) (Figure S3), respectively. Their structures were easily elucidated as aloenin aglycone and aloin A (Figure 4) by comparison of the HPLC retention time with authentic compounds (Figures S2 and S4) [18,19].

Compound **2** was obtained as a brownish amorphous powder. Its molecular weight was determined by HRESIMS as 652 (*m*/*z* 651.1793 (M − H)^−^, calc. as C_33_H_31_O_14_, 651.1714; *m*/*z* 675.1681 (M + Na)^+^, calc. as C_33_H_32_O_14_Na, 675.1690) with an unsaturation degree of 18 (Figures S5–S7). In the ^1^H-NMR (Figure S8) spectrum of **2** (Table 1), the AABB coupling system of *δ*_H_ 7.43 (2H, d, *J* = 8.6 Hz, H-2‴, 6‴) and *δ*_H_ 6.76 (2H, d, *J* = 8.6 Hz, H-3‴, 5‴) was characteristic of a 1,4-disubstituted benzene group. The four aromatic protons at *δ*_H_ 6.47 (1H, d, *J* = 2.0 Hz, H-6′), *δ*_H_ 6.63 (1H, d, *J* = 2.0 Hz, H-4′), *δ*_H_ 6.16 (1H, m, H-5), and *δ*_H_ 6.17 (1H, m, H-7) indicated the presence of two tetrasubstituted benzene rings. In addition, one methoxy group at *δ*_H_ 2.45 (3H, s, 4′–COCH_3_) and two olefinic protons at *δ*_H_ 7.62 (1H, d, *J* = 15.9 Hz, H-7‴) and *δ*_H_ 6.31 (1H, d, *J* = 15.9 Hz, H-8′′′) were observed. The ^13^C-NMR (Figure S9) and the DEPT (Figures S10 and S11) spectra of **2** (Table 1) showed 33 carbon signals attributed to one methyl, three sp^3^ methylene, sixteen methine (ten olefinic and six aliphatic), and thirteen quaternary carbons (three carbonyls, ten sp^2^ including five oxygenated). The NMR signals of *δ*_C_ 61.0 (C-6″), *δ*_C_ 73.2 (C-3″), *δ*_C_ 74.8 (C-2″), *δ*_C_ 77.1 (C-5″), and an anomeric carbon *δ*_C_ 98.9 (C-1″) together with the proton at *δ*_H_ 5.19 (1H, d, *J* = 8.0 Hz, H-1″) suggested the presence of a *β*-d-glucopyranoside.

**Figure 5:**
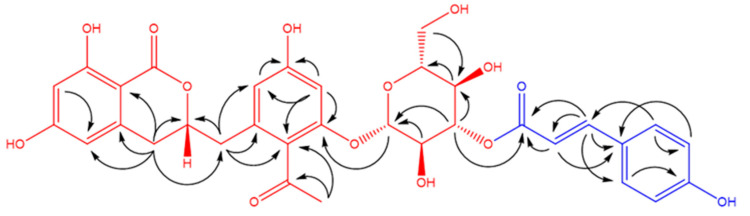
Key HMBC correlations of **2**.

The HMBC (Figure S13) correlations shown in Figure 5 of COCH_3_/COCH_3_, COCH_3_/C-2′, H-4′/C-5′, H-9/C-1′, C-3, H-4/C-3, C-4a, C-8a, and C-9 in combination with the HSQC (Figure S12) correlations and the chemical shifts of *δ*_C_ 164.2 (C-6), *δ*_C_ 164.8 (C-8), *δ*_C_ 166.8 (C-1), *δ*_C_ 123.8 (C-2′), *δ*_C_ 156.3 (C-3′), and *δ*_C_ 159.5 (C-5′) indicated the presence of feralolide [20]. Moreover, the HMBC correlations of H-1″/C-3′ confirmed the linkage pattern between feralolide and glucopyranoside. A careful examination of these NMR data suggested the presence of the moiety of feralolide-3′-*O*-*β*-d-glucopyranoside [21] drawn in red and the moiety of *p*-coumaric acid [22] drawn in blue in Figure 5. The HMBC correlation of H-3″/C-9‴ suggested that the red and the blue parts were connected via C-3″–*O*–C-9‴. Consequently, the structure of **2** was identified and named as aloeacone.

### 2.3. Effects of the Ligands on the Enzymatic Activity of α-Glucosidase

The effects of the ligands fished out on the enzymatic activity of α-glucosidase were measured, which were reflected as the percentage of decrease (inhibition ratio) or increase (maximal effect ratio) of α-glucosidase activity. Aloin A (**3**) inhibited α-glucosidase with an inhibition ratio of 49.0 ± 0.2% (IC_50_ value of 1.29 mM), the result of which is in accordance with the previous study [10]. The positive control, acarbose, showed the inhibitory activity of 61.6%. On the contrary, compounds **1** and **2** showed agonist potentials against α-glucosidase with the maximal effect ratios of 26.4 ± 0.8% and 17.8 ± 0.3%, respectively. This is the first report on the agonists of α-glucosidase from *A. vera* extract. It is presumably because the co-existence of an inhibitor and agonists that resulted in the light inhibition effect of S_5_ was 4.7 ± 0.5%.

### 2.4. Molecular Docking Studies

**Figure 6:**
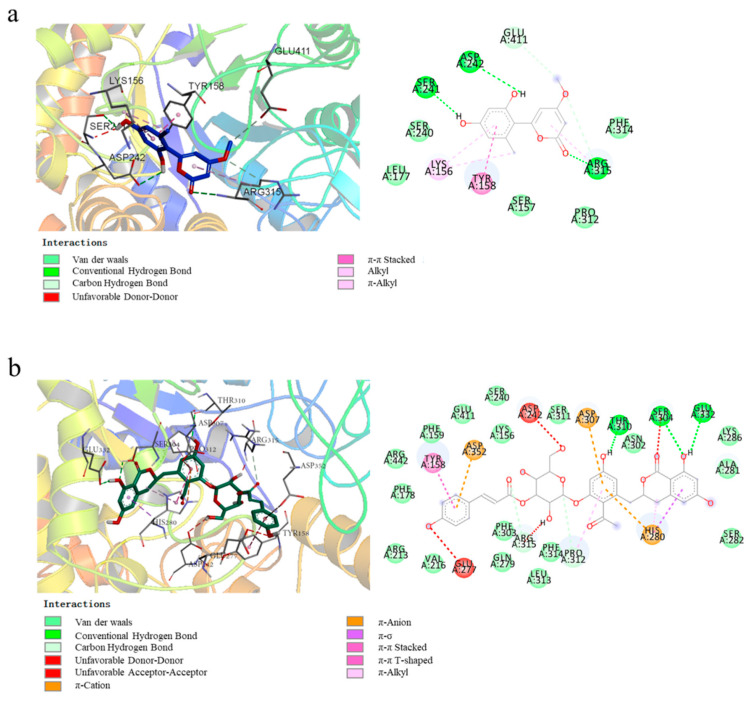

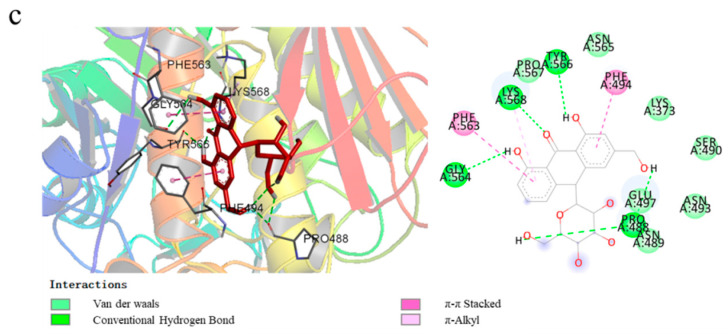
2D and 3D molecular docking graphs of (**a**) compounds **1**, (**b**) compound **2** and (**c**) compound **3** with α-glucosidase.

Molecular docking has become an important technology in computer-aided drug research [23]. This method uses docking to explore the interactions between small molecules and binding pockets of proteins to predict binding patterns and affinity [24]. In this study, the three ligands were docked with α-glucosidase, and the results are shown in Figure 6. The lowest binding free energies were −7.5 kcal/mol for compound **1**, −10.2 kcal/mol for compound **2,** and −8.2 kcal/mol for compound **3**, respectively.

The 2D and the 3D computational binding modes between compound **1** and α-glucosidase are illustrated in Figure 6a. It could be found that three stable hydrogen bonds were formed between **1** with Arg 315, Asp 242, and Ser 241 of the enzyme, and the formation of unstable hydrogen bonds was associated with Glu 411 and Arg 315. Moreover, the amino acid residues Lys 156 and Arg 315 bonded to **1** by alkyl and π–alkyl interactions, and Tyr 158 bonded to **1** via π–π stacked interaction. As shown in Figure 6b, three conventional hydrogen bonds were formed between the phenolic hydroxyl groups of **2** with Ser 304, Thr 310, and Glu 332 of α-glucosidase. Besides, the amino acid residues His 280, Tyr 158, and Pro 312 interacted with the benzene ring of **2** by π–σ, π–alkyl, π–π stacked, and π–π T-shaped interactions. In particular, some ionic bonds (π–cation and π–anion) formed via amino acid residues Asp 352, Asp 307, and His 280 were observed in the graph. In the case of compound **3** (Figure 6c), five stable hydrogen bonds between **3** and α-glucosidase were formed via Gly 564, Lys 568, Tyr 566, Pro 488, and Glu 497. Besides, Phe 563 and Phe 494 interacted with the aromatic ring of **3** through π–π T-shaped interaction. These results suggested that, unlike compound **3,** the α-glucosidase binding sites of **1** and **2** had some amino acid residues in common differing from that of **3**.

## 3. Materials and Methods

### 3.1. Materials and Chemicals

*Aloe vera* was purchased from a drug store of Yumintang Chinese Medicine Corporation (Chengdu, China). α-glucosidase (EC.3.2.1.20, from *Saccharomyces cerevisiae*) was purchased from Sigma-Aldrich (St. Louis, MO, USA). *p*-nitrophenyl α-d-glucoside (*p*-NPG) was purchased from Shanghai Yuanye Bio-Technology Co., Ltd. (Shanghai, China). Acarbose was purchased from Psaitong (Beijing, China). Ethanol, acetonitrile (ACN), glutaraldehyde aqueous solution (50%, GA), formic acid (FA), and dimethyl sulfoxide (DMSO) were purchased from Chengdu Kelong Chemical Reagent Factory (Chengdu, China). (3-Aminopropyl)trimethoxysilane (APTMS) and tetraethyl orthosilicate (TEOS) were purchased from TCI (Tokyo, Japan). Methanol used for HPLC was of high chromatographic grade (JT Baker, Phillipsburg, NJ, USA), and an ultra-purified (UP) water purification (18.25 MΩ) system (Chengdu, China) was used for the HPLC solvent system. All other chemicals, solvents, and reagents were of high analytical grade.

### 3.2. Apparatus and Instruments

HPLC analysis was performed by a Shimadzu LC-20AD equipped with a binary pump and a DAD detector (Waltham, MA, USA). HPLC-MS/MS analysis was performed on a waters ACQUITY system coupled with a triple-quadrupole mass spectrometer (Xevo™, Waters, and Milford, PA, USA). Fourier transform infrared spectra (FT-IR) were recorded in KBr by PerkinElmer (Waltham, MA, USA) FTIR spectrophotometer. Cyclic voltammetry and differential pulse voltammetry were recorded by an electrochemical workstation (CV and DPV; Gaoss Union EC500, China). Thermo Scientific Varioskan Flash equipped with a 96-well microplate (Thermo, Waltham, MA, USA) was used for α-glucosidase inhibition assay.

### 3.3. Preparation of α-Glucosidase Functionalized MNPs

Firstly, MNPs with terminal aldehyde groups were synthesized following a previously reported procedure with minor modification [25]. Briefly, under a nitrogen atmosphere, 2.0271 g of FeCl_3_•6 H_2_O and 0.7407 g of FeCl_2_•4 H_2_O (molar ratio = 1:2) were dissolved in 250 mL deionized water, and 25% ammonium hydroxide was added into the system until the pH value reached 9. After 30 min of reaction, the MNPs were separated by an external magnet and washed with water and ethanol subsequently. The MNPs were then suspended in 150 mL of ethanol containing 400 μL of TEOS, the pH value of which was adjusted to 9 by 25% ammonium hydroxide, and the mixture was stirred for 5 h. The core-shell structured SiO_2_-MNPs obtained were collected and washed subsequently with water and ethanol before coated with a layer of amino groups with 2 mL of APTMS in 90 mL ethanol containing 1 mL water at 35 °C overnight to obtain the NH_2_-MNPs. After that, the NH_2_–MNPs were dispersed in 150 mL water and stirred with 25 mL 50% glutaraldehyde for 3 h to terminate the MNPs with aldehyde groups (CHO-MNPs). Secondly, the solubilized α-glucosidase was covalently immobilized on CHO-MNPs by crosslinking. The CHO-MNPs (3 mg) were dispersed in 1 mL of phosphate buffer solution (PBS) (50 mM, pH 6.8) containing α-glucosidase (1 mg) to incubate for 6 h at 30 °C. The α-glucosidase immobilized MNP (AG-MNP) was separated by magnet and washed three times with PBS (50 mM, pH 6.8) and finally suspended in PBS to store at 4 °C for future use.

### 3.4. Characterization of α-Glucosidase Functionalized MNPs

FTIR was used to characterize the surface modification of the MNPs. The electrochemical method could be used for CHO-MNPs and AG-MNPs analysis in which immobilization of the MNPs on the electrode usually resulted in the change of current response [26]. In this work, by using carbon as a working/counter electrode and Ag/AgCl as a reference electrode for the electrochemical workstation, the cyclic voltammograms (CV) and the differential pulse voltammograms (DPV) of the nano-composites were measured in PBS (10 mM, pH 7.0) containing 5 mM K_3_[Fe(CN)_6_] and 0.1 M KCl. Operationally, CHO-MNPs and AG-MNPs were suspended, respectively, in PBS at a concentration of 10 mg/mL, and then 10 μL suspension liquid was added into a screen-printed carbon electrode (SPCE) reaction cell for the CV and the DPV measurements. The potential range was set from −0.1 to 0.6 V at a scan rate of 50 mV/s.

### 3.5. Preparation of Extract of A. vera

Two grams of dry leaves of *A. vera* were powdered and extracted with 40 mL of 70% methanol at r.t. by ultrasonication for 30 min. Since the leaves were air-dried, the chemical constituents present in latex and gel of fresh leaves were extracted together with other compounds in this step. The extraction solution was filtered with 0.22 μm filtration membranes, and the filtrate was concentrated to dryness and diluted in PBS (50 mM, pH 6.8), denoted as S_0_ for the following experiment.

### 3.6. Fishing of α-Glucosidase’s Ligands from A. vera

A total of 1 mL of S_0_ was added into a 1.5 mL Eppendorf tube containing 10 mg of AG-MNPs. The mixture was shaken vigorously for 40 min, and the AG-MNPs adsorbed with ligands of the enzyme was separated magnetically by an external magnet and washed four times using PBS (50 mM, pH 6.8) to remove the non-specifically adsorbed compounds. In the end, the AG-MNPs composite was eluted with 300 μL of 50% can, which was denoted as S_5_. Then, S_0_ and S_5_ were analyzed by the following HPLC and HPLC-MS. The HPLC condition was as follows: the column was Agilent (Palo Alto, CA, USA) ZORBAX SB–C18 (250 × 4.6 mm, 5 μm); UV detection wavelength was 254 nm; the mobile phase consisted of solvent A (0.1%, *v*/*v*, formic acid/water) and solvent B (methanol); the gradient elution was 40–70% B at 0–25 min, 70–100% B at 25–27 min, and 100% B at 27–32 min; and the flow rate was 0.8 mL/min.

Compound **2** was isolated from S_5_ by preparative HPLC using a semi-preparation column Cosmosil (Kyoto, Japan) 5C_18_-MS-II (10.0 mm I.D × 250 mm). The elution condition was similar to the above except the flow rate was 2.0 mL/min, and the elution gradient was 40–70% B at 0–60 min. Finally, 5 mg of compound **2** was obtained from 12 mg of S_5_.

### 3.7. Enzymatic Activity Assay of the Enzyme’s Ligands

The activity assay of the fished-out ligands against α-glucosidase was carried out on 96-well microtiter plates following a method reported previously [27]. The ligands and the positive control, acarbose, were dissolved in PBS (50 mM, pH 6.8) with 5% DMSO, while α-glucosidase and *p*-NPG were dissolved in PBS (50 mM, pH 6.8) alone. In total, 50 μL of α-glucosidase (2 U/mL) was incubated with an equal volume of the test compound solution (0.5 mg/mL) at 37 °C for 10 min. Then, 100 μL of the *p*-NPG solution (3 mM) was added and then incubated at 37 °C for 20 min. In the end, 100 μL of Na_2_CO_3_ (0.2 M) was added to the mixture to terminate the reaction. The amount of the product, *p*-nitrophenol (*p*-NP), was detected by ultraviolet absorption spectrum at 405 nm. All assays were performed in triplicates, and the α-glucosidase inhibition rate (I%) was calculated according to the following formula:

I% = [1 − (T_R_ − T_B_)/(C_R_ − C_B_)] × 100% (1)

where T_R_ represents the absorbance of test reaction; T_B_ represents the absorbance of test blank; C_R_ represents the absorbance of control reaction; and C_B_ represents the absorbance of control blank.

### 3.8. Molecular Docking Study

Molecular docking was used to study the binding mode between the ligands and α-glucosidase through Autodock vina 1.1.2 (Scripps Research, CA, USA) [28]. Since the three-dimensional structural information of α-glucosidase from *Saccharomyces cerevisiae* was not available in the RCSB Protein Data Bank (http://www.rcsb.org, accessed on 16 August 2021), the crystal structure of isomaltase (PDB code: 3A4A) from *S. cerevisiae,* which has the highest (84%) sequence similarity to α-glucosidase, was used for the docking study [29,30]. The 3D structures of the ligands were depicted by ChemDraw 14.0 and Chem3D 14.0 (PerkinElmer, Waltham, MA, USA), and the docking input files were generated using Auto Dock Tools 1.5.6 (Scripps Research, CA, USA) package [31]. The binding mode with the lowest free energy predicted by Autodock vina was selected as the best. The binding mode was analyzed by PyMoL 1.7.6 software (Schrödinger, NY, USA), and the diagrams were simulated using Discovery Studio (Neotrident, Beijing, China).

## 4. Conclusions

In this work, three ligands of α-glucosidase were specifically extracted from *A. vera* extract by using AG-MNPs, which were identified as aloenin aglycone (**1**), aloeacone (**2**), and aloin A (**3**). Among them, compound **2** was found to be a new natural product with an agonist effect on α-glucosidase. This is the first report on the co-existence of an inhibitor and agonists of α-glucosidase in *A. vera*, and it is of help to understand the anti-diabetes activity of the plant. It is also noted that the ligand fishing method proposed in this work can not only reveal the enzyme’s ligands present in the complex mixture of the herbal extractions but is also able to enrich certain amounts of those, ensuring effective isolation and preparation of the target compound.

## Figures and Tables

**Table 1:** ^1^H-NMR (600 MHz) and ^13^C-NMR (150 MHz) data of **2** (*δ* in ppm, *J* in Hz, methanol-*d*_4_).

2					
No.	*δ*_H_	*δ*_C_	No.	*δ*_H_	*δ*_C_
1		166.8	1″	5.19, d, 8.0	98.9
3	4.65, m	79.8	2″	3.71, m	74.8
4	2.76, m	32.1	3″	5.13, m	73.2
4a		141.7	4″	3.55, m	70.0
5	6.16, m	106.6	5″	3.55, m	77.1
6		164.2	6″	3.97, dd, 12.0, 2.0	61.0
7	6.17, m	100.8		3.78, dd, 12.0, 5.4	
8		164.8	1‴		125.9
8a		100.1	2‴	7.43, d, 8.6	129.8
9	2.98, dd, 13.8, 7.2	37.6	3‴	6.76, d, 8.6	115.4
	2.84, dd, 13.8, 5.0		4‴		159.9
1′		136.8	5‴	6.76, d, 8.6	115.4
2′		123.8	6‴	7.43, d, 8.6	129.8
3′		156.3	7‴	7.62, d, 15.9	145.8
4′	6.63, d, 2.0	101.1	8‴	6.31, d, 15.9	113.6
5′		159.5	9‴		166.8
6′	6.47, d, 2.0	112.0			
COCH_3_		205.5			
COCH_3_	2.45, s	31.9			

## Data Availability

The data presented in this study are available on request from the corresponding author.

## Supplementary Materials

The following are available online, Figure S1: MS spectra of **1**, Figure S2: HPLC-UV analysis of (**a**) aloenin aglycone isolated from *Aloe vera*; (**b**) solution of ligands, S_5_, Figure S3: MS spectrum of **3**, Figure S4: HPLC-UV analysis of (**a**) *Aloe vera* extract solution, S_0_; (**b**) aloin A isolated from *Aloe vera*; (**c**) reference substance of aloin, Figure S5: MS spectra of **2**, Figure S6: HRESIMS spectrum of **2** (positive ion mode), Figure S7: HRESIMS spectrum of **2** (negative ion mode), Figure S8: ^1^H-NMR (600 Hz) spectrum of **2** in methanol-*d*_4_, Figure S9: ^13^C-NMR (150 Hz) spectrum of **2** in methanol-*d*_4_, Figure S10: DEPT 135° spectrum of **2** in methanol-*d*_4_, Figure S11: DEPT 90° spectrum of **2** in methanol-*d*_4_, Figure S12: HSQC spectrum of **2** in methanol-*d*_4_, Figure S13: HMBC spectrum of **2** in methanol-*d*_4_.

## Sample Availability

Samples of the compounds are not available from the authors.
