# Supplementary material for: Fishing of α-Glucosidase’s Ligands from Aloe vera by α-Glucosidase Functionalized Magnetic Nanoparticles
Source: Molecules. 2021 Sep 26;26(19):5840. doi: 10.3390/molecules26195840 (PMC8510290; doi:10.3390/molecules26195840)
Supplement: Supplementary file 1 [file molecules-26-05840-s001.zip › molecules-1378357-supplementary.pdf]

Supplementary Material: Fishing of α-glucosidase's ligands from *Aloe vera* by α-glucosidase functionalized magnetic nanoparticles

Hao Yuan, Hong Wan, Yi-kao Hu, Qiang Chang, Emmanuel Ayodeji Ayeni, Xun Liao

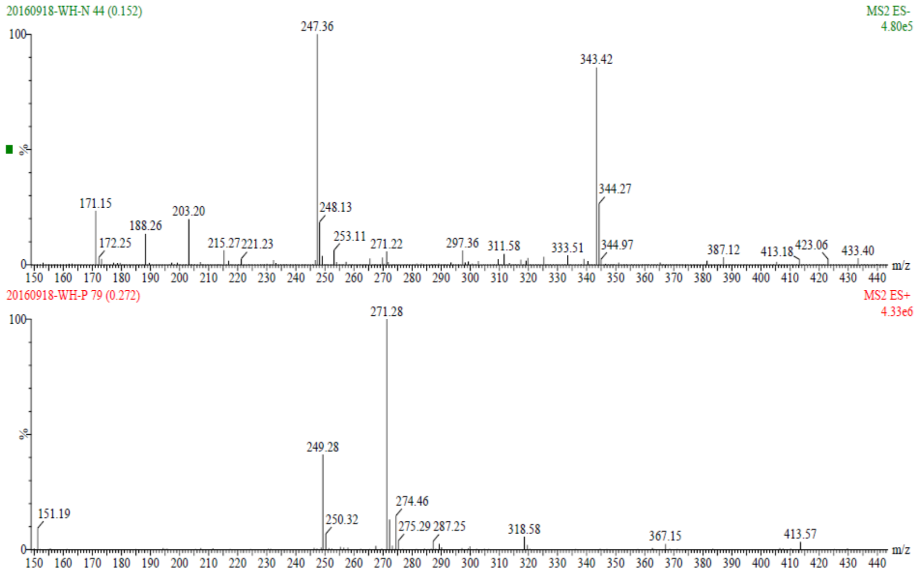

Figure S1. MS spectra of **1**.

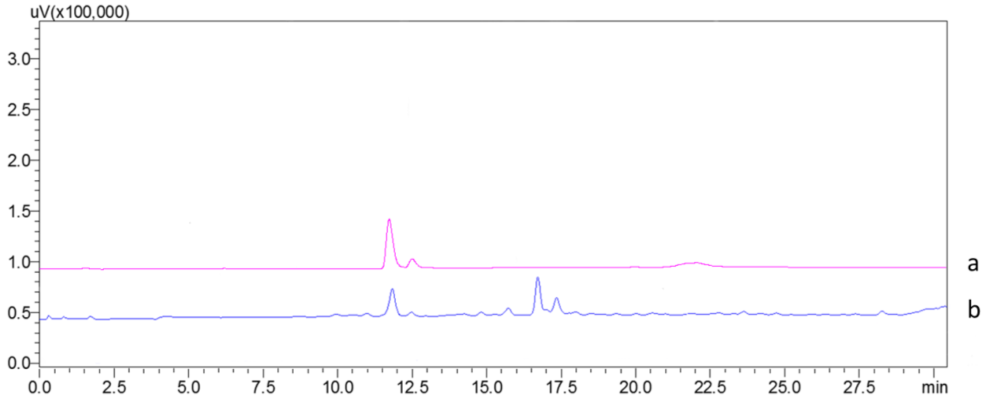

Figure S2. HPLC-UV analysis of (a) aloenin aglycone isolated from *Aloe vera*; (b) solution of ligands, S_5.

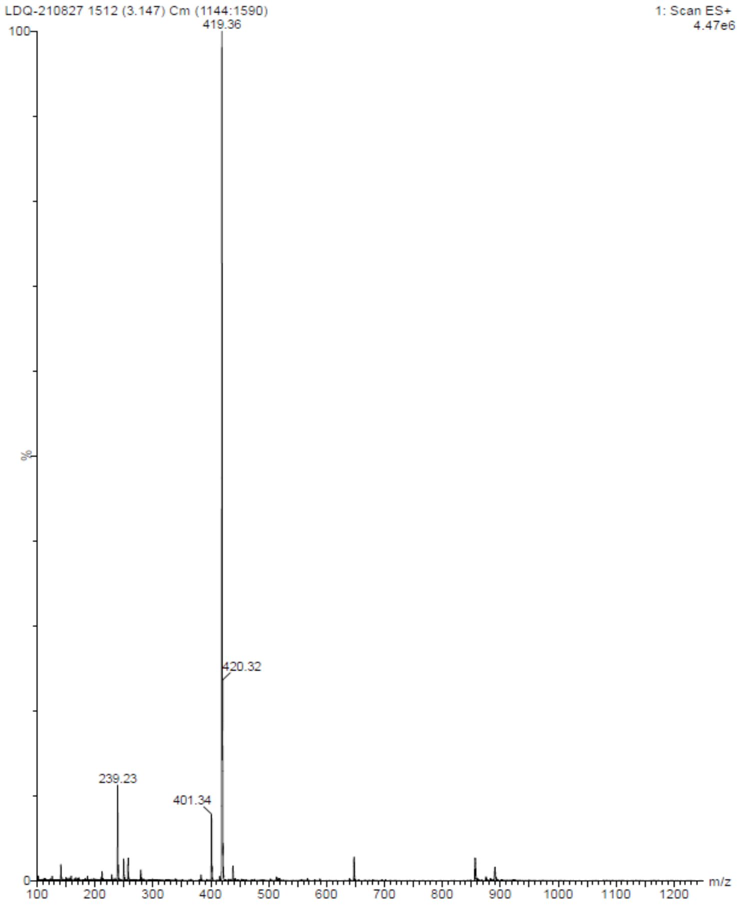

Figure S3. MS spectrum of **3**.

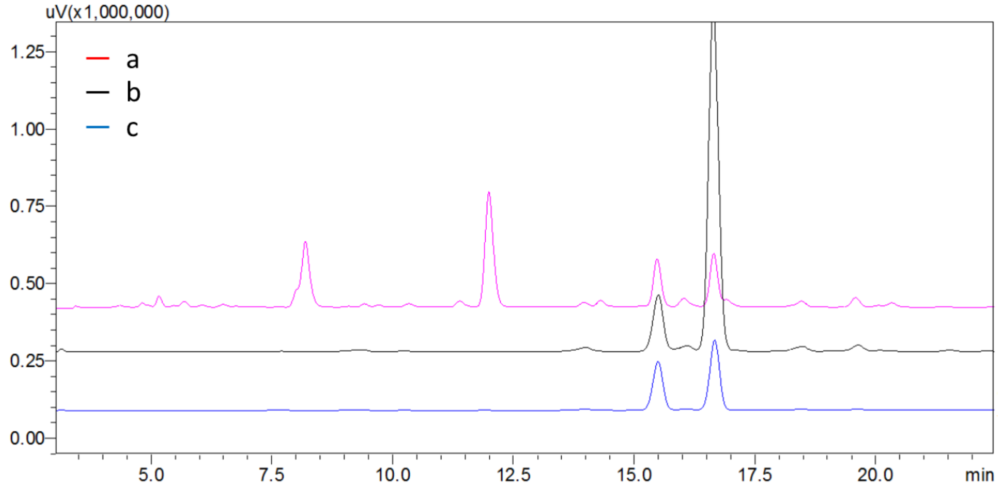

Figure S4. HPLC-UV analysis of (a) *Aloe vera* extract solution, S_0; (b) aloin A isolated from *Aloe vera*; (c) reference substance of aloin.

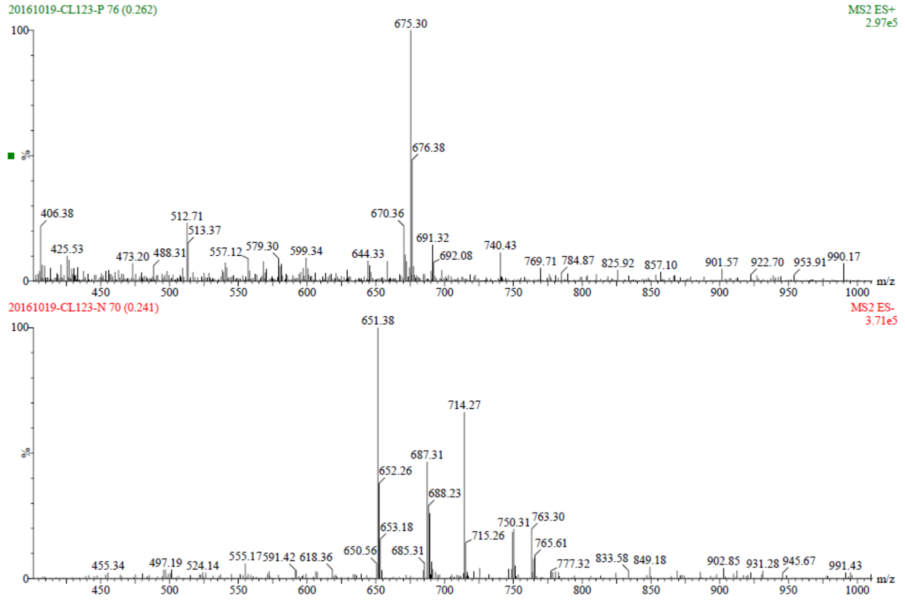

Figure S5. MS spectra of **2**.

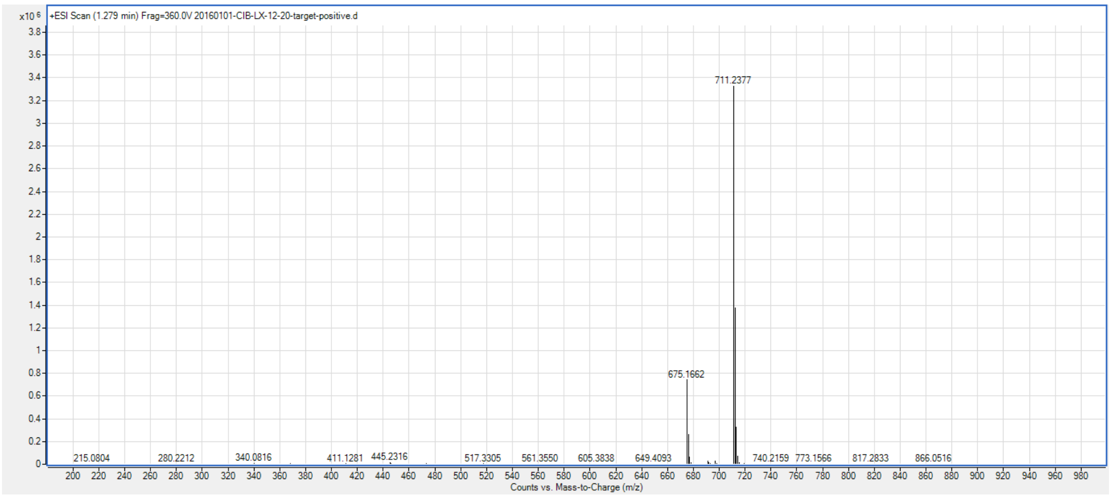

Figure S6. HRESIMS spectrum of **2** (positive ion mode).

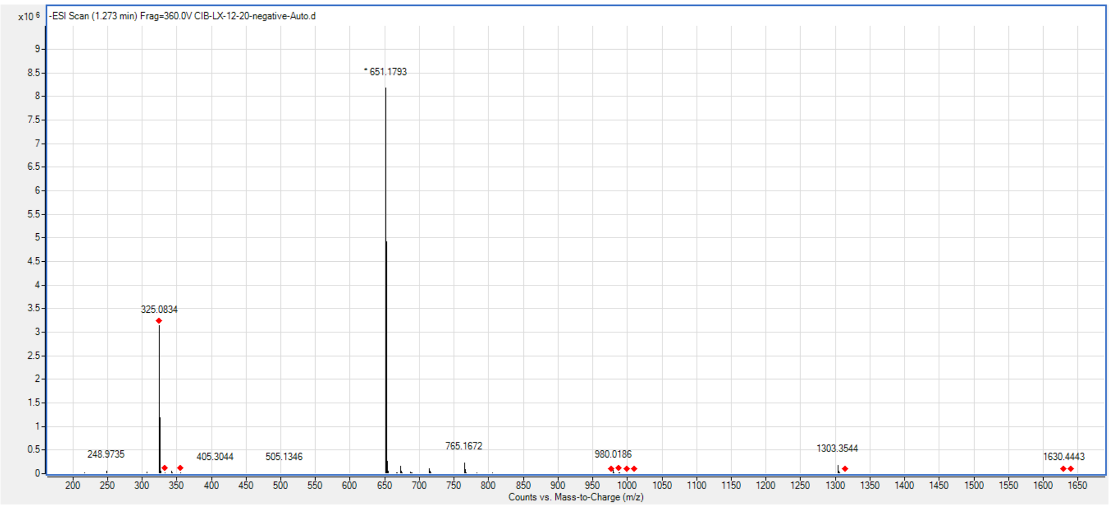

Figure S7. HRESIMS spectrum of **2** (negative ion mode).

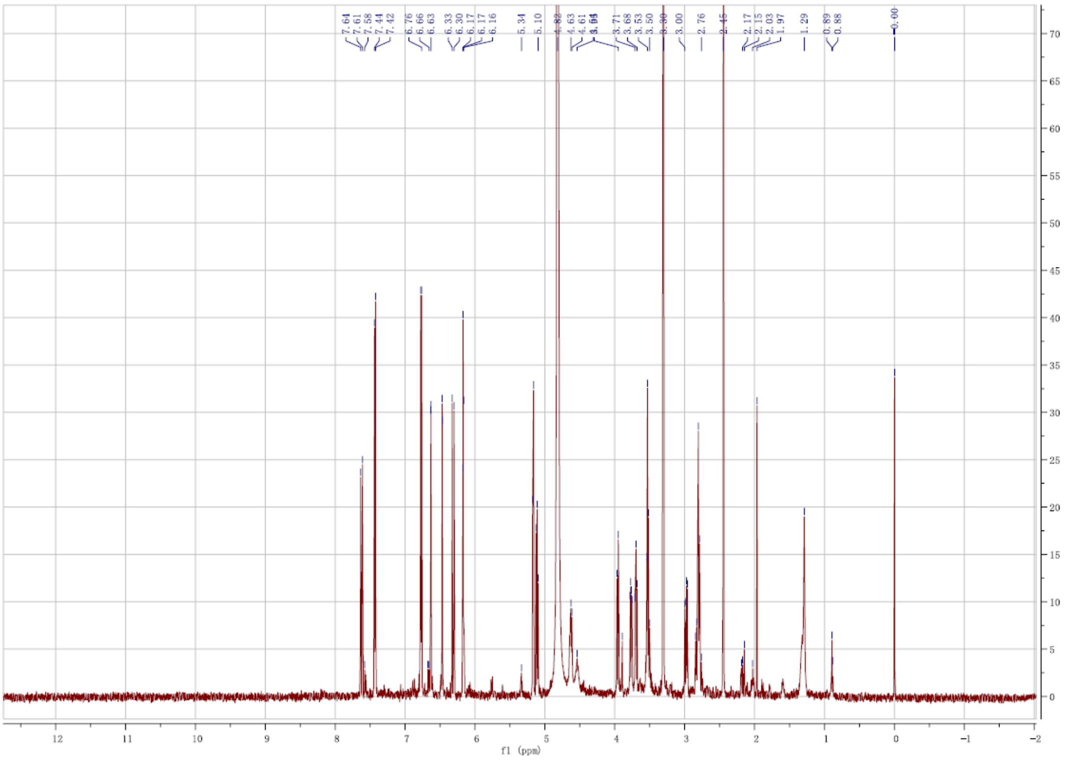

Figure S8. ^{1}H NMR (600 Hz) spectrum of **2** in methanol-d_4.

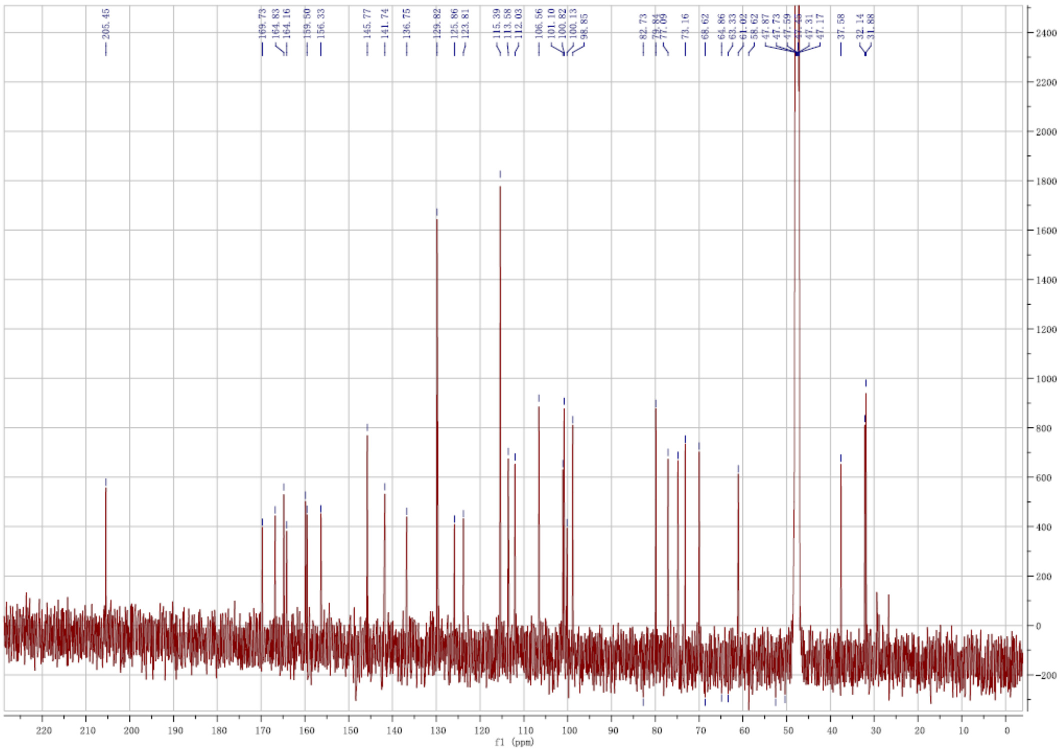

Figure S9. ^{13}C NMR (150 Hz) spectrum of **2** in methanol-d_4.

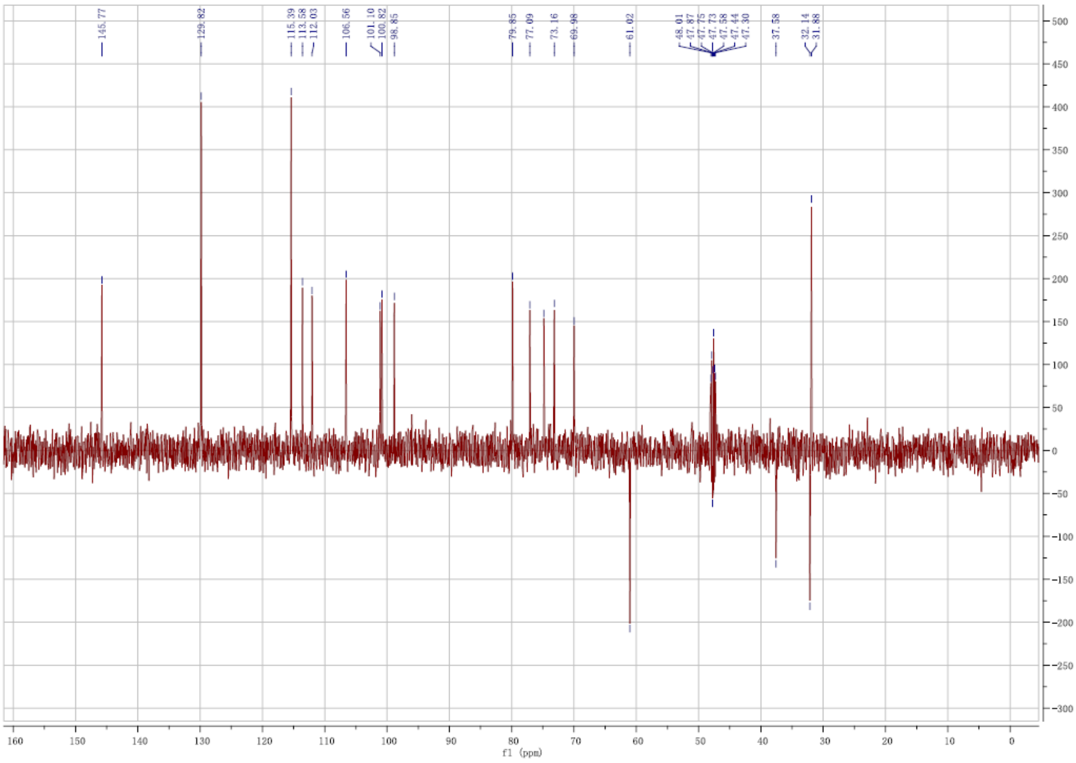

Figure S10. DEPT 135 °spectrum of **2** in methanol-d_4.

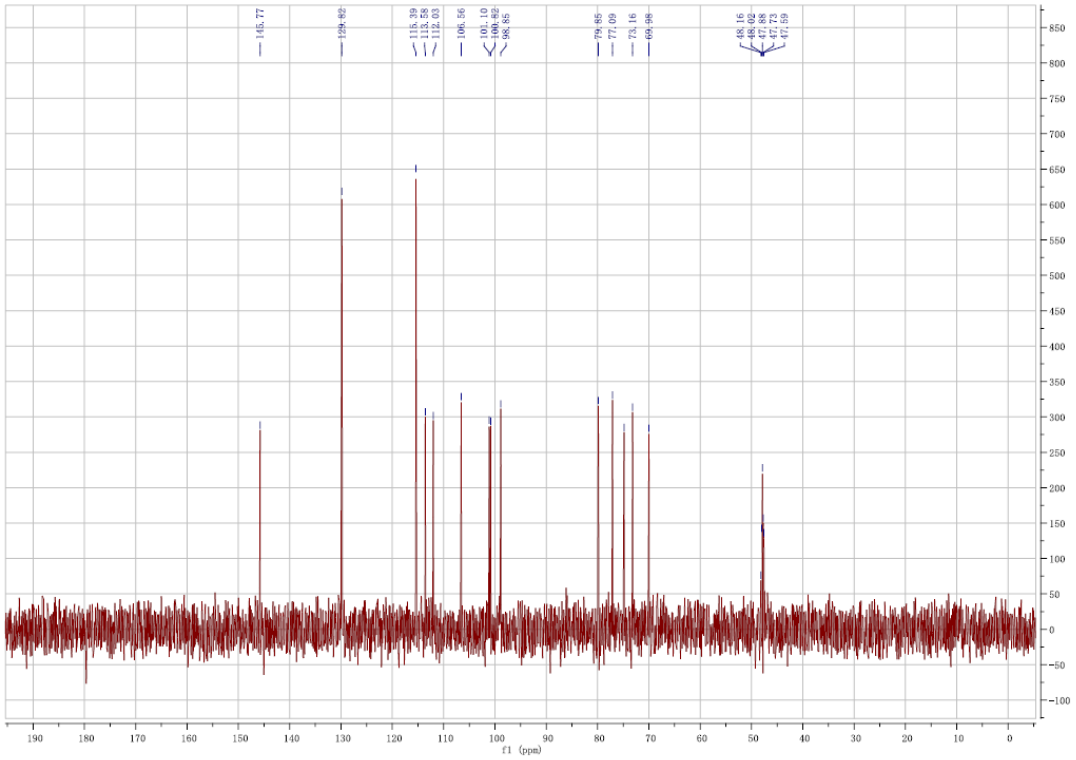

Figure S11. DEPT 90 °spectrum of **2** in methanol-d_4.

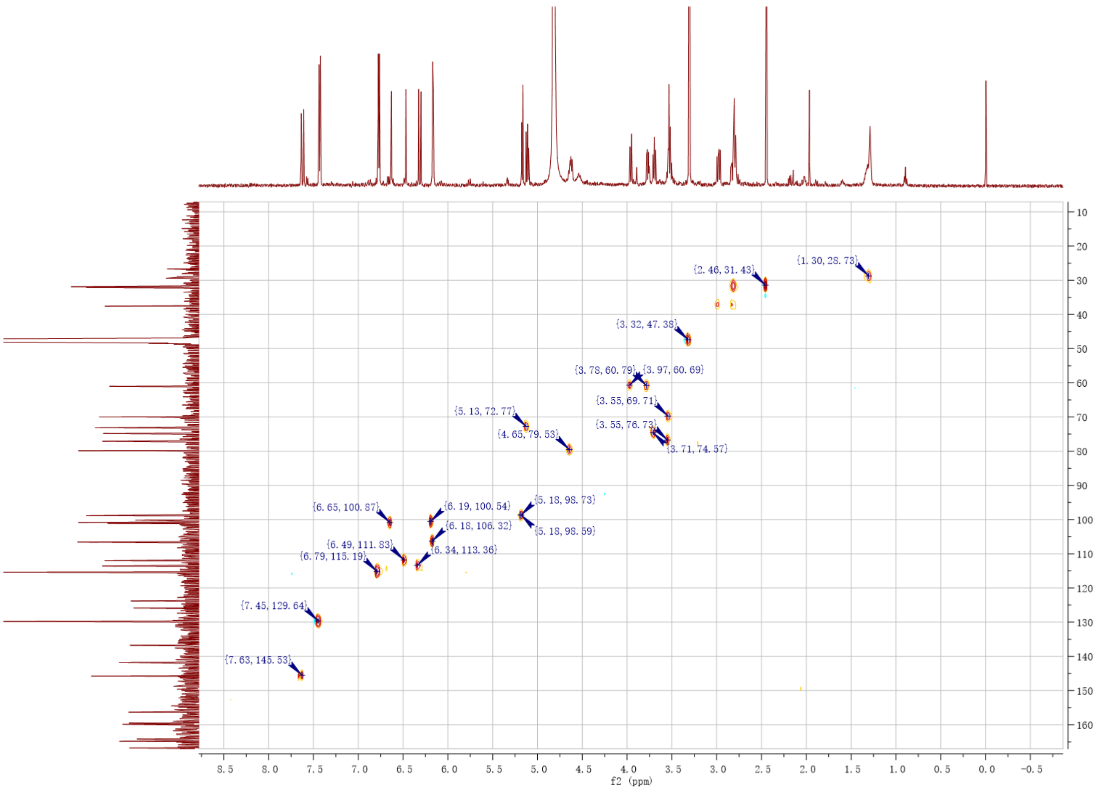

Figure S12. HSQC spectrum of **2** in methanol-d_4.

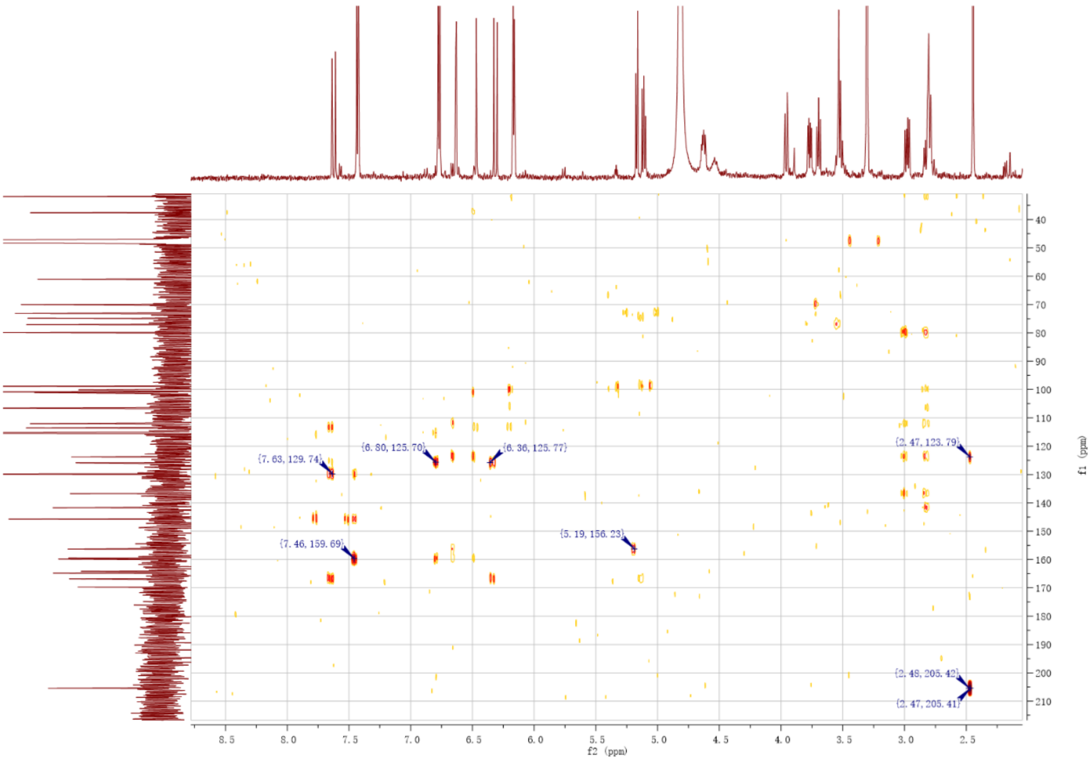

Figure S13. HMBC spectrum of **2** in methanol-d_4.
